# C3H/HeNSlc mouse with low phospholipid transfer protein expression showed dyslipidemia

**DOI:** 10.1038/s41598-023-40917-9

**Published:** 2023-08-24

**Authors:** Misato Kobayashi, Fumi Kanbe, Reika Ishii, Hiroki Tsubouchi, Kana Hirai, Yuki Miyasaka, Tamio Ohno, Hiroaki Oda, Saiko Ikeda, Hirokazu Katoh, Kenji Ichiyanagi, Akira Ishikawa, Atsushi Murai, Fumihiko Horio

**Affiliations:** 1 Department of Nutritional Sciences, Nagoya University of Arts and Sciences, 57 Takenoyama, Iwasaki-Cho, Nisshin, Aichi 470-0196 Japan; 2 Department of Animal Sciences, Graduate School of Bioagricultural Sciences, Nagoya University, Aichi, Japan; 3 Division of Experimental Animals, Graduate School of Medicine, Nagoya University, Aichi, Japan; 4 Department of Applied Biosciences, Graduate School of Bioagricultural Sciences, Nagoya University, Aichi, Japan; 5 Department of Life Studies and Environmental Science, Nagoya Women’s University, Aichi, Japan

**Keywords:** Metabolic disorders, Biochemistry, Lipids

## Abstract

High serum levels of triglycerides (TG) and low levels of high-density lipoprotein cholesterol (HDL-C) increase the risk of coronary heart disease in humans. Herein, we first reported that the C3H/HeNSlc (C3H-S) mouse, a C3H/HeN-derived substrain, is a novel model for dyslipidemia. C3H-S showed hypertriglyceridemia and low total cholesterol (TC), HDL-C, and phospholipid (PL) concentrations. To identify the gene locus causing dyslipidemia in C3H-S, we performed genetic analysis. In F2 intercrosses between C3H-S mice and strains with normal serum lipids, the locus associated with serum lipids was identified as 163–168 Mb on chromosome 2. The phospholipid transfer protein (*Pltp*) gene was a candidate gene within this locus. *Pltp* expression and serum PLTP activity were markedly lower in C3H-S mice. *Pltp* expression was negatively correlated with serum TG and positively correlated with serum TC and HDL-C in F2 mice. Genome sequencing analysis revealed that an endogenous retrovirus (ERV) sequence called intracisternal A particle was inserted into intron 12 of *Pltp* in C3H-S. These results suggest that ERV insertion within *Pltp* causes aberrant splicing, leading to reduced *Pltp* expression in C3H-S. This study demonstrated the contribution of C3H-S to our understanding of the relationship between TG, TC, and PL metabolism via PLTP.

## Introduction

There is a strong inverse correlation between the risk of coronary heart disease and plasma levels of high-density lipoprotein cholesterol (HDL-C)^1,2^. Low-density lipoprotein (LDL) is an atherogenic lipoprotein found in human plasma. Lowering of low-density lipoprotein cholesterol (LDL-C) with statin drugs has shown to reduce atherogenic cardiovascular events; however, even with these drug-induced benefits, low HDL-C levels remain a risk factor for cardiovascular events^3^. The anti-atherogenic function of HDL is mainly attributed to reverse cholesterol transport (RCT), which represents cellular free cholesterol efflux from the peripheral tissues to the liver. Further, the anti-inflammatory and antioxidant effects of HDL-C possibly contribute to its anti-atherogenic function as well^4^. Hypertriglyceridemia is associated with other lipid abnormalities, including low HDL-C levels, presence of small dense LDL particles, atherogenic triglyceride (TG)-rich remnants, and insulin resistance^5^. A clinical study suggested that high TG levels combined with low HDL-C levels contribute to the risk of coronary heart disease^3^.

Naturally occurring mutant mice and genetically manipulated mice have been used as experimental models for human cardiovascular disease and dyslipidemia^6^. In mice fed standard chow, 80–90% of the serum total cholesterol (TC) was present in the HDL fraction. Mice lack cholesteryl ester transfer protein, which mediates the transfer of cholesteryl esters from HDL to VLDL/LDL, and the transfer of TG in the opposite direction. Despite these differences between mice and humans, observations obtained from experiments on mice have uncovered the genes and mechanisms involved in lipid metabolism and atherogenesis in humans^6^.

We found that C3H/HeNSlc (C3H-S) mice, obtained from SLC Japan, Inc. (Shizuoka, Japan), are a useful mouse model for hypertriglyceridemia and low HDL-C levels. Other C3H/HeN-derived substrains C3H/HeNCrlCrlj (C3H-C) and C3H/HeNJcl mice were obtained from Charles River Laboratories Japan Inc. (Yokohama, Japan) and CLEA Japan (Shizuoka, Japan), respectively. C3H-C and C3H/HeNJcl mice from the two vendors did not show dyslipidemia. Further, C57BL/6N-derived substrains obtained from different vendors also showed phenotypic differences among the substrains^7^. Although their substrains were closely related, they contained single nucleotide polymorphisms (SNPs)^8^. These reports suggest that the mutation occurs within the gene that regulates serum levels of both TG and HDL-C in C3H-S mice.

This study aimed to characterize the dyslipidemia symptoms and identify the genes causing dysregulation of TG and HDL-C metabolism in C3H-S mice. We used genetic analysis to search for the causative gene of dyslipidemia in C3H-S mice. Using F2 intercrosses between C3H-S mice and the control strains, C57BL/6J (B6) and C3H-C, we detected chromosomal regions (Chr2: 163–168 Mb) that were strongly associated with high serum levels of TG and low levels of TC, HDL-C, and phospholipids (PL). Furthermore, we investigated the relationship between serum phospholipid transfer protein (PLTP) expression/activity and serum lipid concentration, and the results suggested that *Pltp* is a causative gene for dyslipidemia in C3H-S mice.

## Results

### C3H-S mouse is a novel model for dyslipidemia

**Figure 1:**
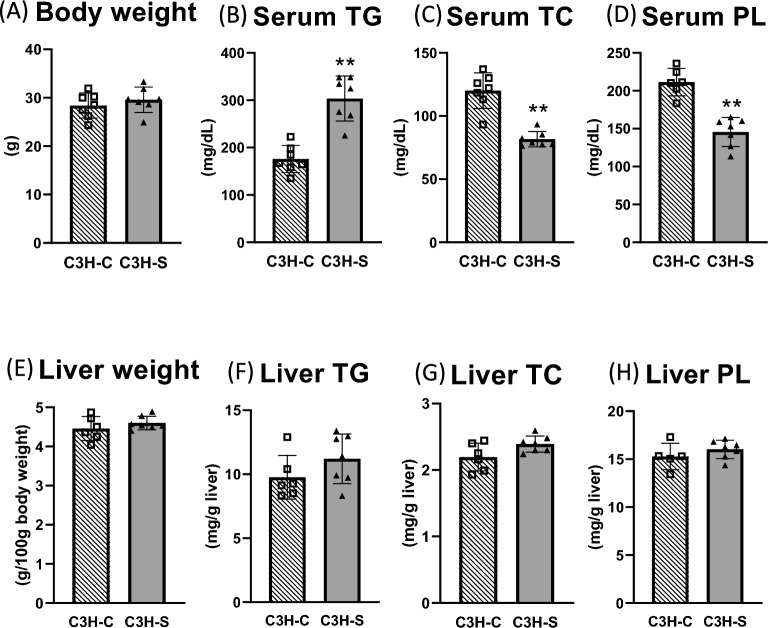
Body weight, serum and liver lipids concentrations in male C3H-C mice and male C3H-S mice. (**A**) Body weight, (**B**) Serum TG, (**C**) TC, and (**D**) PL were measured in male mice after 4 h fasting at 15 weeks of age. (**E**–**H**) Liver was obtained in male mice after 4 h fasting at 10 weeks of age. C3H-C (n = 6–7), C3H-S (n = 7–8). Welch’s tests were performed for the phenotypic parameters. **P < 0.01.

**Figure 2:**
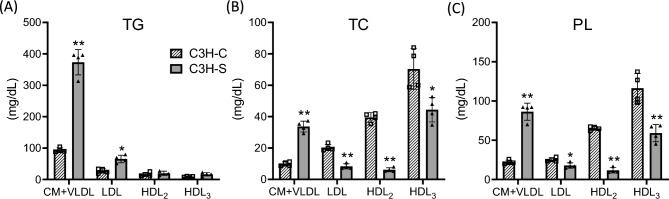
Serum lipids concentrations of lipoproteins in male C3H-C and C3H-S mice. (**A**) TG, (**B**) TC, and (**C**) PL concentrations of serum lipoproteins in male C3H-C and C3H-S mice (9–12 weeks of age, n = 4). CM + VLDL (d < 1.006 g/mL), LDL (d = 1.006–1.060 g/mL), HDL_2_ (d = 1.060–1.085 g/mL) and HDL_3_ (d = 1.085–1.163 g/mL) were fractionated by ultracentrifugation. *P* values were obtained from Welch’s tests. *P < 0.05, **P < 0.01.

C3H-C and C3H-S mice were supplied as C3H-HeN strains by different vendors. At 15 weeks of age, there was no difference in body weight between male C3H-C and C3H-S mice (Fig. 1A). Male C3H-S mice showed markedly higher levels of serum TG and lower levels of serum TC and PL than did male C3H-C mice after 4 h of fasting (Fig. 1B–D). In contrast, the liver weight and liver TG, TC, and PL concentrations in C3H-S mice did not differ from those in C3H-C mice (Fig. 1E–H). C3H-S mice did not exhibit liver lipid accumulation. The VLDL-TG and LDL-TG concentration in C3H-S mice was higher than those in C3H-C mice (Fig. 2A). In cholesterol-rich lipoproteins (LDL and HDL), the cholesterol and PL concentrations in C3H-S mice were markedly lower than those in C3H-C mice (Fig. 2B,C). These data indicate that C3H-S mice are a novel model for dyslipidemia, especially hypertriglyceridemia.

### Detection of a dyslipidemia-associated locus in an F2 intercross between C3H-S mice and B6 mice

**Figure 3:**
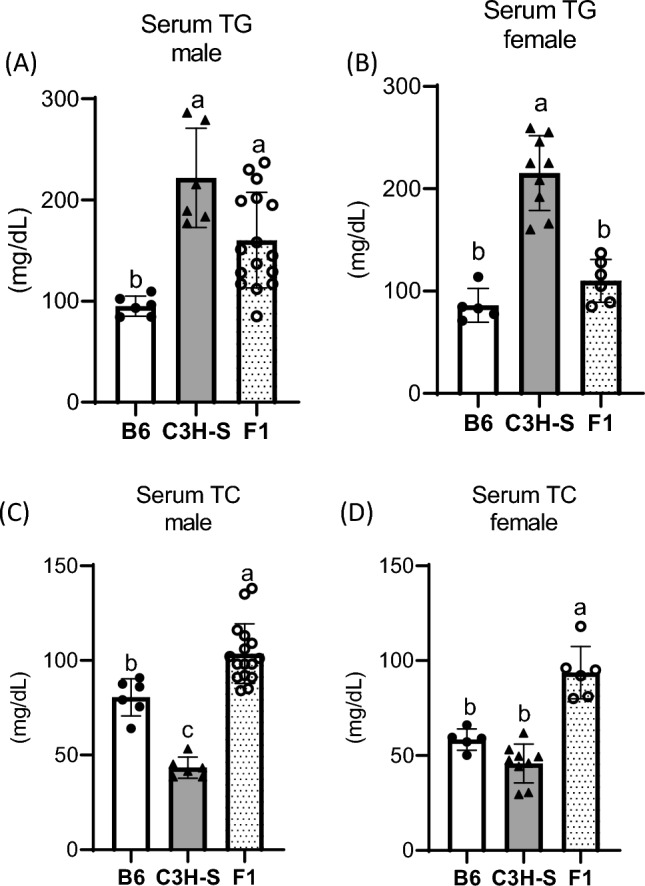
Serum lipids concentrations in C3H-S, B6, and F1 mice at 8–9 weeks of age. (**A**, **B**) Serum TG and (**C**, **D**) TC concentration of C3H-S, B6, and (C3H-S × B6)F1 mice at 8–9 weeks of age. C3H-S (male n = 6, female n = 9), B6 (male n = 6, female n = 5), F1 (male n = 16, female n = 6–7). ^a–c^Means without common letters are significantly different by Tukey’s test (*P* < 0.05).

We performed genetic analysis of the F2 intercross to identify the chromosomal regions responsible for dyslipidemia in C3H-S mice. B6 mice were chosen as the parental strain of F2 mice. Both male and female B6 mice showed lower serum TG concentrations than male and female C3H-S mice, respectively (Fig. 3A,B). The serum TG levels of male F1 mice were higher than those of B6 mice, but those of female F1 mice were comparable to those of B6 mice. The serum TC concentrations in male B6 mice were higher than those in male C3H-S mice, but those in male and female F1 mice were significantly higher than those in B6J and C3H-S mice (Fig. 3C,D).

**Figure 4:**
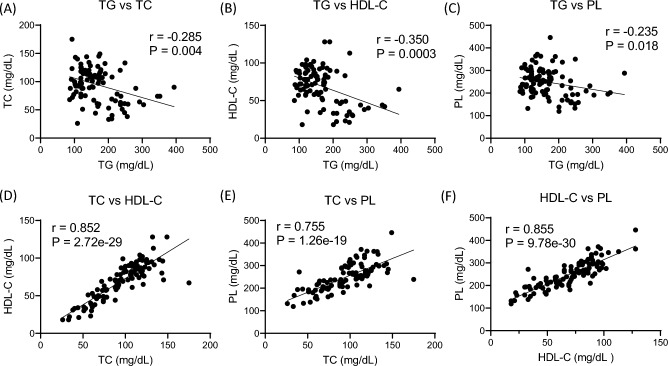
The correlation of serum lipids concentrations in (C3H-S × B6)F2 mice. (**A**) TG vs TC, (**B**) TG vs HDL-C, (**C**) TG vs PL, (**D**) TC vs HDL-C, (**E**) TC vs PL, (**F**) HDL-C vs PL. (C3H-S × B6)F2 mice (n = 100). Spearman's correlations were performed for each pair of the serum lipids.

F2 mice were obtained by intercrossing (C3H-S × B6)F1 mice. The serum lipid concentrations of male (C3H-S × B6)F2 mice (n = 100) were assayed, and the correlation between each serum lipid is shown in Fig. 4. Serum TG concentrations were negatively correlated with serum TC, HDL-C, and PL concentrations in the F2 mice. In contrast, serum TC vs. HDL-C, TC vs. PL, and HDL-C vs. PL concentrations showed a positive correlation.

We labelled the top 15 mice with serum TG concentrations as the high-TG group and the bottom 15 mice as the low-TG group in the F2 mice. First, the genomic DNA of high- or low-TG groups was pooled as one sample for genotype determination. To detect the chromosomal region associated with serum TG concentration, genotyping analysis using pooled genomic DNA of the high- or low-TG groups in F2 mice was performed; this is known as bulk segregation analysis, which is an efficient screening method^9,10^. Using microsatellite markers, we detected differences in the band pattern of electrophoresis on chromosome 2 between the high- and low-TG groups (Supplementary Fig. S1A). Genotyping analysis using pooled DNA suggested that the locus controlling TG is near D2Mit226 (163.194 Mb) on mouse chromosome 2. Further, to identify the loci associated with serum TG and other serum lipids (TC, HDL-C, and PL), genotyping analysis was performed using genomic DNA from F2 mice (n = 100). Table 1 shows the mean values of the serum parameters for each genotype on chromosome 2 in F2 mice. From rs27325969 (chr.2:155.21 Mb) to rs51710768 (chr.2:173.53 Mb) in the chromosomal region, the mean value of serum TG in F2 mice with C3H-S homozygotes was higher than that in F2 mice with the B6 allele (B6/B6 and B6/C3H-S) (Table 1). In contrast, the mean values of serum TC, HDL-C, and PL in F2 mice with C3H-S homozygotes were lower than those in F2 mice with the B6 allele. Table 2 shows the chromosomal markers associated with serum lipid concentrations in the (C3H-S × B6)F2 mice. The highest coefficients of determination for serum TG, TC, HDL-C, and PL levels were detected at the same chromosomal position (rs13459398; chr2:164.85 Mb) (Table 2). The rs13459398 SNP is located at the exon 6 in *Pltp* gene. The locus of chr.2:164.85 Mb explained 45.9, 60.0, 64.7, and 41.0% of the variations in the concentration of the serum lipids TG, TC, HDL-C, and PL, respectively. These results revealed that the locus near chr2:164.85 Mb controls not only serum TG concentration but also serum TC, HDL-C, and PL concentrations.

**Table 1:** The values of serum lipids in each genotype in male (C3H-S × B6)F2 mice.

Marker	Trait	(C3H-S × B6)F2		
(position)	(mg/dL)	B6/B6	B6/C3H-S	C3H-S/C3H-S
rs27325969 (155.21 Mb)		n = 24	n = 46	n = 30
	TG	137 ± 32^a^	143 ± 42^a^	**220 ± 69**^b^
	TC	104 ± 18^a^	**108 ± 27**^a^	68 ± 23^b^
	HDL-C	76 ± 12^a^	**82 ± 21**^a^	46 ± 19^b^
	PL	**262 ± 43**^a^	273 ± 56^a^	202 ± 54^b^
D2Mit226 (163.19 Mb)		n = 22	n = 50	n = 28
	TG	145 ± 30^a^	133 ± 39^a^	**227 ± 68**^b^
	TC	**110 ± 20**^a^	105 ± 29^a^	61 ± 18^b^
	HDL-C	**82 ± 16**^a^	79 ± 17^a^	40 ± 15^b^
	PL	**274 ± 48**^a^	269 ± 51^a^	189 ± 42^b^
rs13459398 (164.85 Mb)		n = 22	n = 50	n = 28
	TG	133 ± 30^a^	143 ± 36^a^	**231 ± 66**^b^
	TC	105 ± 20^a^	**111 ± 20**^a^	59 ± 16^b^
	HDL-C	79 ± 16^a^	**83 ± 15**^a^	38 ± 12^b^
	PL	269 ± 48^a^	**275 ± 50**^a^	187 ± 40^b^
rs27295296 (166.08 Mb)		n = 22	n = 49	n = 29
	TG	142 ± 30^a^	133 ± 36^a^	**229 ± 66**^b^
	TC	**111 ± 20**^a^	105 ± 20^a^	60 ± 18^b^
	HDL-C	**83 ± 16**^a^	79 ± 15^a^	40 ± 14^b^
	PL	**276 ± 48**^a^	269 ± 51^a^	189 ± 40^b^
rs51710768 (173.53 Mb)		n = 23	n = 53	n = 24
	TG	159 ± 38^a^	136 ± 63^a^	**207 ± 56**^b^
	TC	104 ± 21^a^	**105 ± 26**^a^	65 ± 25^b^
	HDL-C	76 ± 18^a^	**81 ± 21**^a^	45 ± 21^b^
	PL	261 ± 49^a^	**276 ± 59**^a^	196 ± 41^b^

Mean ± SD. Bold indicates the maximum values of each trait at the respective marker.

^a,b^Means without common letters are significantly different by Tukey’s (parametric) or Dunn’s (non-parametric) multiple comparison test (*P* < 0.05).

**Table 2:** The coefficient of determinations between the chromosomal markers and serum lipids concentrations in the (C3H-S × B6)F2 mice.

Marker (position)	Trait	Coefficient of determination (R^2^)	*P*-value
rs27325969 (155.21 Mb)	TG	0.352	7.48e−10
	TC	0.356	4.12e−10
	HDL-C	0.428	1.68e−12
	PL	0.264	3.40e−7
D2Mit226 (163.19 Mb)	TG	0.407	9.92e−12
	TC	0.515	5.97e−16
	HDL-C	0.566	2.56e−18
	PL	0.382	7.55e−11
rs13459398 (164.85 Mb)	TG	**0.459**	**1.16e**−**13**
	TC	**0.600**	**4.79e**−**20**
	HDL-C	**0.647**	**1.13e**−**22**
	PL	**0.410**	**7.57e**−**12**
rs27295296 (166.08 Mb)	TG	0.452	2.10e−13
	TC	0.574	1.01e−18
	HDL-C	0.628	1.56e−21
	PL	0.406	1.07e−11
rs51710768 (173.53 Mb)	TG	0.170	1.18e−4
	TC	0.328	4.13e−9
	HDL-C	0.338	2.11e−9
	PL	0.249	9.51e−7

The correlation between each allele at loci on chromosome 2 and serum lipids concentrations of each mouse were analyzed. Statistical analyses for data of (C3H-S × B6)F2 mice (n = 100) were performed using JMP Pro version 13.2.0 software.

Bold indicates the strongest correlation of each trait.

The rs13459398 (Chr2: 164,852,656) SNP is located at Exon 6 in *Pltp* gene.

### Confirmation of dyslipidemia-associated locus on chromosome 2 in an F2 intercross between C3H-S and C3H-C mice

To confirm the locus associated with serum lipids, we performed an F2 analysis between C3H-S and C3H-C mice. In male (C3H-S × C3H-C)F2 mice (n = 41), serum TG concentration was negatively correlated with serum PL concentration (Supplementary Fig. S2). Serum TC concentration was positively correlated with HDL-C and PL concentrations. The correlations between serum lipids in (C3H-S × C3H-C)F2 mice were similar to those in (C3H-S × B6)F2 mice. The mean values of the serum parameters for each genotype on chromosome 2 are shown in Supplementary Table S1. At chr2:163.08–168.52 Mb, the mean values of serum TG in F2 mice with C3H-S homozygotes were higher than those in F2 mice with C3H-C allele (C3H-C/C3H-C and C3H-C/C3H-S) (Supplementary Table S1). The mean values of serum TC, HDL-C, and PL in F2 mice with C3H-S homozygotes were lower than those in F2 mice with C3H-C allele. The highest coefficient of determination (0.733, *P* = 1.28e−11) for serum TG concentrations was detected at the SNP_168.52 Mb (chr.2:168.52 Mb) (Supplementary Table S2). This locus explained 73.3% of the variation in serum TG concentrations in F2 mice. Correlation analysis in (C3H-S × C3H-C)F2 mice showed that the loci SNP_163.08 Mb and SNP_168.52 Mb were strongly associated with TG, TC, HDL-C, and PL concentrations. This result indicates that a major gene controlling serum lipid levels exists on chromosome 2. Genetic analyses of both (C3H-S × B6)F2 and (C3H-S × C3H-C)F2 revealed a locus responsible for dyslipidemia in C3H-S mice on chr.2: 163–168 Mb.

### Gene expression and activity of PLTP in C3H mice were markedly low

**Figure 5:**
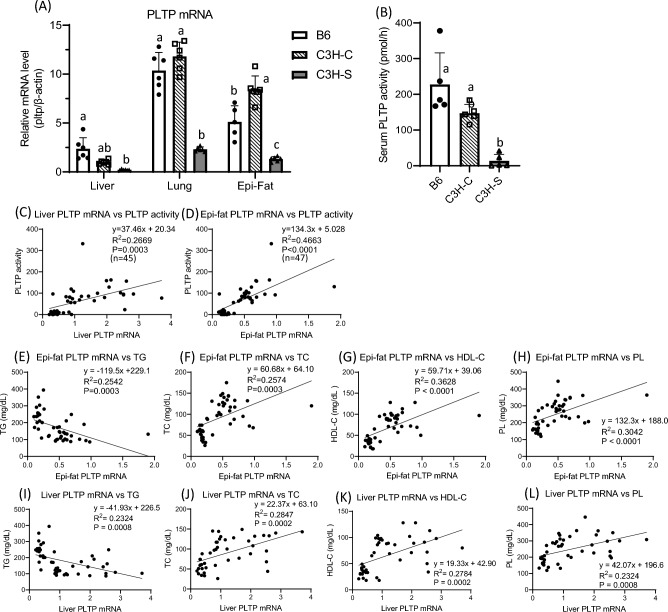
The PLTP expression in tissues and serum PLTP activity, and the regression between PLTP mRNA levels and serum lipids. (**A**) PLTP mRNA levels in liver, lung and epididymal fat (Epi-fat) among male B6, C3H-C and C3H-S mice (n = 5–6). The mRNA level of C3H-C in liver was set to 1. mRNA levels were expressed as relative values of C3H-C in liver. (**B**) Serum PLTP activities among male B6, C3H-C and C3H-S mice (n = 5). ^a–c^Means without common letters are significantly different by Tukey test (*P* < 0.05). (**C**, **D**) Sigle linear regression between PLTP mRNA (relative mRNA levels) and PLTP activities (pmol/h) in select F2 mice. (**E**–**H**) Single linear regression between PLTP mRNA in Epi-fat and serum lipids in select F2 mice. (**I**–**L**) Single linear regression between PLTP mRNA in liver and serum lipids in select F2 mice.

We explored candidate genes associated with serum lipoprotein metabolism within the chromosomal region from chr.2:163 Mb to chr.2:168 Mb. We found that the *Pltp* gene (164.839–164.858 Mb) was the most likely candidate gene for explaining dyslipidemia in C3H-S mice. *Pltp* mRNA levels in the lungs were the most abundant among the three tissues, and the levels in epididymal fat were higher than those in the liver (Fig. 5A). In the lungs and epididymal fat, *Pltp* mRNA levels in both C3H-C and B6 mice were higher than those in C3H-S mice. Serum PLTP activity was low in C3H-S mice, which coincided with the trend of *Pltp* mRNA levels (Fig. 5A,B). Regression analyses between *Pltp* mRNA and PLTP activity in F2 mice indicated that the low level of serum PLTP activity was due to the low level of *Pltp* mRNA (Fig. 5C,D). *Pltp* expression in epididymal fat was more strongly associated with serum PLTP activity than in the liver. Further, to determine the relationship between serum lipid levels and *Pltp* expression or PLTP activity, we assayed the *Pltp* mRNA levels and serum PLTP activity in (C3H-S × B6)F2 mice (Fig. 5E–L). The top 10 and bottom 10 of each serum lipids of F2 mice were selected and assayed. *Pltp* expressions in epididymal fat and liver were negatively correlated with serum TG concentration, accounting for approximately 25 and 23% of total variation in the serum TG (R^2^ = 0.2542, R^2^ = 0.2324, respectively) (Fig. 5E,I). In contrast, *Pltp* mRNA expressions in both tissues were positively correlated with serum TC, HDL-C, and PL concentrations (Fig. 5F–H,J–L). Furthermore, in (C3H-S × C3H-C)F2 mice, *Pltp* expression accounted for approximately 41, 32, and 34% of total variation in serum TG, TC, and HLD-C, respectively (Supplementary Fig. S3).

### Insertion of the endogenous retrovirus (ERV) sequence affected transcripts of the Pltp gene in C3H-S mice

**Figure 6:**
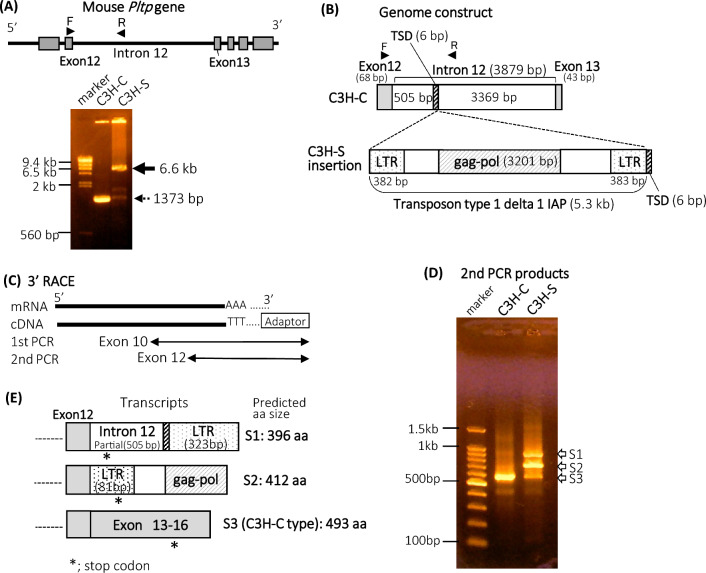
The sequence of endogenous retrovirus inserted into the intron 12 of the *Pltp* gene altered mRNA construction in the C3H-S mice. (**A**) The intron 12 of the *Pltp* gene was amplified from C3H-C and C3H-S mouse genomic DNA using the forward (F) and reverse (R) primers (arrowhead). F and R primers were located on exon 12 and intron 12, respectively. The predicted PCR product size was 1373 bp (dashed arrow). (**B**) Insertion of the ERV into intron 12 of the *Pltp* gene. *gag-pol* putative gag-pol protein gene, *IAP* intracisternal A-type particle, *LTR* Long terminal repeat, *TSD* Target site duplication. (**C**) The cloning of *Pltp* cDNA 3′ ends. 3′RACE was performed using primers on exon 9 (1st PCR) and exon 12 (2nd PCR). (**D**) The predicted size of 2nd PCR product was S3 (C3H-C type). (**E**) The cDNA construction and predicted amino acid (aa) size of S1, S2, and S3. *stop codon. Original gels in (**A**) and (**D**) are presented in Supplementary Fig S5.

Whole-genome sequencing analyses revealed that SNPs between C3H-C and C3H-S mice were not detected in the *Pltp* gene or within 500 kb of it. However, a part of sequence of intron 12 in C3H-S mice could not be obtained by next-generation sequencing. To determine the sequence, we amplified the genomic region containing a part of intron 12 using specific primers (Fig. 6A). The PCR product size (approximately 6.6 kb) obtained from C3H-S mice was markedly greater than that obtained from C3H-C mice (predicted size = 1.3 kb). DNA sequencing of PCR products revealed that the target site duplication (TSD) sequence (TAGTGC) exists within intron 12 of *Pltp* in both C3H-C and C3H-S mice (Fig. 6B). In C3H-S mice, the 5300 bp fragment was inserted following the TSD (submitted in the DDBJ database as LC726248). The end of the insertion also had a 6 bp sequence of TSD. The remaining fragment had a sequence similar to that of the mouse transposon type 1 delta 1 intracisternal A-type particle (1Δ1 IAP). The 1Δ1 IAP had an open reading frame encoding a gag-pol fusion protein. Both end sequences (382 and 383 bp) of 5.3 kb were very similar to the long terminal repeat (LTR) of the mouse ERV (IAPLTR1_Mm).

We performed sequence analyses of transcripts containing exon 12 to 3′-ends (Fig. 6C). The expected (S3) and unexpected (S1 and S2) transcript sizes were detected in C3H-S mice (Fig. 6D). Although the S3 transcript coincided with that of C3H-C mice, the S1 and S2 transcripts contained partial sequences of the transposon 1Δ1 IAP (Fig. 6E). The S1 transcript contained exon 12, followed by the partial sequence of intron 12 (505 bp), TSD, and LTR of ERV. The S2 transcript contained exon 12, followed by an inserted sequence (LTR and gag-pol of ERV) without the partial sequence of intron 12. The amino acid sequences and sizes predicted from the S1 and S2 transcripts differed from those of S3, which was similar to that of the wild-type C3H-C transcript (Fig. 6E, Supplementary Fig. S4).

## Discussion

In this study, we found that C3H-S mice are a new spontaneous dyslipidemia mouse model. C3H-S mice showed high levels of serum TG and low levels of HDL-C compared with normal strains, C3H-C and B6 mice. We identified the locus controlling serum lipid concentrations in C3H-S mice using (C3H-S × B6)F2 and (C3H-S × C3H-C)F2 mice. F2 analyses showed that serum TG, TC, HDL-C, and PL concentrations were associated with a locus at 163–168 Mb on mouse chromosome 2 (Table 2, Supplementary Table S2). The C3H-S homozygote at the locus near 163–168 Mb showed higher TG and lower TC, HDL-C, and PL levels than the C3H-C homozygote (Table 1, Supplementary Table S1). We searched for the most likely candidate gene, and found *Pltp*, which is reportedly involved in HDL metabolism^11^.

PLTP plays an important role in the transfer of PLs between lipoprotein particles and modulation of HDL size and composition^12^. A previous study using PLTP knockout mice showed markedly decreased levels of HDL-containing lipids and apolipoproteins, demonstrating that PLTP modulates the maintenance of HDL levels^12^. These reports suggest that low *Pltp* expression in C3H-S and F2 mice lead to low serum HDL-C concentrations (Figs. 2, 5G,K, Supplementary Fig. S3C).

Genomic DNA sequencing analysis revealed that the ERV sequence was inserted into intron 12 of *Pltp* in C3H-S mice (Fig. 6B). The ERV sequence within the *Pltp* gene was identified as 1Δ1 IAP. IAP sequences are highly abundant in the mouse genome^13^. C3H strains have numerous IAP insertional mutations compared to other strains, the reasons for which are currently unknown^14^. The insertion of ERVs within genes affects transcript processing, such as splicing and polyadenylation, which alters gene expression^15^. It has been reported that the ERVs insertions within an intron disrupt normal splicing^16,17^. Small amounts of normal transcripts and normal proteins of the gene in which the ERVs was inserted can be detected. The aberrant splicing of gene is promoted by ERV elements, leading to the synthesis of various aberrant or chimeric transcripts^18^. In this study, the 1Δ1 IAP insertion within the *Pltp* gene caused an alteration of the transcripts (S1 and S2 in Fig. 6E) and mutations in the amino acid sequences of PLTP, which might disrupt the function of the PLTP protein in C3H-S mice (Fig. 6E, Supplementary Fig. S4). Serum PLTP activity decreased in C3H-S mice owing to a decrease in the wild-type transcript of the *Pltp* gene. These findings indicate that ERV insertion affecting *Pltp* expression may cause dyslipidemia in C3H-S mice. The S1 and S2 transcripts are lack of exons 13–16 (Fig. 6E). The S1 and S2 transcripts are chimeric transcripts, which are fusions of exon 12 and intron 12 or part of the ERV sequence of the *Pltp* gene. The S1 and S2 transcripts are translated to 396 amino acid (aa) and 412 aa, respectively. Exon 1–12 of *Pltp* gene is translated into 392 aa. These results suggest that C3H-S mice have residues 1–392 of the wild-type PLTP protein but not residues 393–493. Huuskonen et al*.* reported that the deletion of 50 residues of C-terminal amino acids of human PLTP protein completely abolished secretion and phospholipid transfer activity^19^. The S1 and S2 transcripts of C3H-S may cause reduced serum PLTP activity because they lack the C-terminal residue of PLTP. Although *Pltp* mRNA and PLTP activity in F2 mice showed a strong correlation (Fig. 5C,D), it is not clear whether the low level of serum PLTP activity is due to low levels of PLTP mRNA and/or lack of the C-terminal region of the transcript.

Regarding TG metabolism, our study of C3H-S and F2 mice revealed that low *Pltp* expression was associated with high TG concentrations (Fig. 5E, Supplementary Fig. S3). Lipoprotein profiles showed that VLDL-TG levels in C3H-S mice with low *Pltp* expression were markedly higher than those in C3H-C mice (Fig. 2A). In contrast, PLTP knockout mice did not display any significant changes in serum TG concentration compared to wild-type mice and PLTP^+/−^ mice (C57BL background) fed chow or a high-fat diet^12^. At present, it is unclear why C3H-S mice with low PLTP expression and activity exhibit overt hypertriglyceridemia. However, it is possible that aberrant PLTP protein lacking the C-terminus is produced in C3H-S mice (Fig. 6, Supplementary Fig. S4). Thus, the aberrant PLTP protein in C3H-S mice may affect TG metabolism.

We concluded that *Pltp* is a highly likely causative gene for dyslipidemia in C3H-S mice. *Pltp* expression in C3H-S mice was markedly decreased by the insertion of the ERV sequence. Low serum PLTP activity in C3H-S mice with low *Pltp* expression was associated with hypertriglyceridemia, low serum TC or hypocholesterolemia, and low PL concentrations. This study could not determine whether the insertion of ERV within intron 12 of *Pltp* results in reduced *Pltp* expression, which directly causes dyslipidemia. The deletion of the ERV sequence in C3H-S or knock-in of the ERV sequence in C3H-C would provide direct evidence. This study revealed that PLTP affected serum TG levels. Future studies should investigate the underlying mechanism of serum TG metabolism regulated by the *Pltp* gene. This study expands our understanding of the relationship among TG, cholesterol, and PL metabolism via PLTP.

## Methods

### Animals

#### Parental strains

C3H/HeNCrlCrlj (C3H-C) mice (Charles River Laboratories Japan, Inc.), C3H/HeNSlc (C3H-S) mice (SLC Japan Inc.), and C57BL/6JSlc (B6) mice (SLC Japan Inc.) were purchased and maintained at a controlled temperature of 23 ± 2 °C and humidity of 55 ± 5% with a 12/12-h light/dark cycle. The mice were provided free access to water and a standard CE-2 laboratory diet (CLEA Japan). Blood samples from male C3H-C, C3H-S, and B6 mice were collected from the orbital vein after 4 h of fasting (9:00–13:00); Thereafter, the mice were euthanized via cervical dislocation. Liver, lung, and epididymal fat were collected and immediately frozen.

#### F1 mice

(C3H-S × B6)F1 mice were obtained from crosses between female C3H-S mice (n = 3) and male B6 mice (n = 1). (C3H-S × C3H-C)F1 mice were obtained by crossing female C3H-S mice (n = 3) and male C3H-C mice (n = 1). For the serum lipid assay, blood samples were collected from the tail vein of male F1 mice aged 8–9 weeks after 4 h of fasting. To produce F2 mice, F1 mice were mated at 9 weeks of age.

#### F2 mice

Male (C3H-S × B6)F2 mice (n = 100) and male (C3H-S × C3H-C)F2 mice (n = 41) were obtained from each cross of F1 mice. At 8 weeks of age, blood samples of all F2 mice were collected from the orbital veins after 4 h of fasting. Thereafter, the mice were euthanized via cervical dislocation. Liver and epididymal fat were collected and immediately frozen. Animal care and experimental procedures were approved by the Animal Research Committee of Nagoya University (approval nos. 2017030218 and 2018031315) and were conducted according to the Regulations for Animal Experiments at Nagoya University. All experiments were performed in accordance with the relevant guidelines and regulations as well as the ARRIVE guidelines.

### Analysis of liver and serum lipids

Serum TG, TC, HDL-C, and PL concentrations were measured using the Triglyceride E, Cholesterol E, HDL-cholesterol E, and Phospholipid C-tests (Wako Pure Chemical Industries, Osaka, Japan). Frozen livers (0.3 g) were homogenized in 50 mL chloroform:methanol (2:1), and liver lipids were extracted in organic solvents. The organic extract was used for the measurement of lipids as previously described by Folch et al.^20^. A portion of this extract was dried (TG; 200 μL, TC; 300 μL, and PL; 100 μL), and the hepatic contents of TG, TC, and PL were measured by the Triglyceride E-test, the Cholesterol E-test, and the Phospholipid C-test, respectively.

### Serum lipids concentration of fractionated lipoproteins

At 9–12 weeks of age, blood from C3H-C and C3H-S mice was collected from the orbital vein after 4 h of fasting (9:00–13:00), and the serum was separated via centrifugation at 2400×*g* for 10 min (Model 3700; Kubota, Osaka, Japan). Serum lipoproteins [CM + VLDL fraction (d < 1.006 g/mL), LDL fraction (d = 1.006–1.060 g/mL), HDL_2_ fraction (d = 1.060–1.085 g/mL), and HDL_3_ fraction (d = 1.085–1.163 g/mL)] were fractionated by sequential ultracentrifugation (TL-100 tabletop ultracentrifuge, TLA100.2 rotor; Beckman Instruments Inc., USA)^21–24^. NaCl and NaBr were used for density adjustment. Each fraction was dialyzed using dialysis membrane 8/32 (Sekisui Medical, Co. Ltd., Japan) against PBS buffer, and the dialyzed fractions were used for assays.

### Serum PLTP activity

Serum PLTP activity was measured using a Roar PLTP Activity Assay Kit (Roar Biomedical, New York, NY, USA). Fluorescence was measured using an EnSpire Multimode Plate Reader (PerkinElmer, Inc., Waltham, MA, USA). Serum (3 μL) was mixed with 97 μL of the assay solution (containing PLTP donor particles, assay buffer, and acceptor particles) and incubated at 37 °C for 20 min. The fluorescence intensity was measured at 465 nm excitation/535 nm emission.

### Genotyping and detection of dyslipidemia-associated locus in (C3H-S × B6)F2 mice

Genomic DNA was extracted from the liver using salt-ethanol precipitation. Regarding serum TG concentration, genomic DNA from the top 15 mice or bottom 15 mice in (C3H-S × B6)F2 was pooled as the high-TG or low-TG group DNA, respectively. Pooled samples were prepared by mixing equimolar quantities of DNA from each mouse. To detect the chromosomes associated with dyslipidemia using bulk segregation analysis^9,10^, pooled DNA was used for genotyping 55 markers (Supplementary Table S3). To determine the genotype of each (C3H-S × B6)F2 mouse, primers for microsatellite markers and SNPs were used on chromosome 2 at 11 markers (Supplementary Table S4). Genomic DNA was amplified via PCR using each primer set of microsatellite markers, and the size differences of PCR products were detected with agarose gel electrophoresis (NuSieve 3:1; FMC, Rockland, ME, USA). For SNPs, restriction enzymes were used to digest the PCR products derived from specific alleles. Thereafter, the digests were separated via agarose gel electrophoresis.

### Confirmation of dyslipidemia-associated locus in (C3H-S × C3H-C)F2 mice

To determine the genotype of each (C3H-S × C3H-C)F2 mouse (n = 41), primers for SNPs were used on chromosome 2 at five markers (Supplementary Table S5). These SNPs were selected as genotyping markers from the SNPs detected via whole-genome sequencing analysis.

### Whole-genome sequencing analysis

Genomic DNA of C3H-C and C3H-S mice was extracted via salt-ethanol precipitation. Libraries were prepared using TruSeq DNA PCR version 350. Sequencing was performed using the Illumina NovaSeq 6000 system (Macrogen, Tokyo, Japan). The mapping reference was mm10 for the UCSC (December 2011). The analysis software packages used were Isaac Aligner (ver. 01.15.02.08), Isaac variant caller (ver. 2.0.13), SnpEff (ver. 4.1, GRCm38.81, and dbSNP142), Manta (ver. 0.20.2), and control-FREEC (ver. 6.4). Genome sequencing data were submitted to the SRA (accession no. PRJNA827968).

### Genome DNA sequencing analysis of intron 12 of Pltp gene

The genomic DNA of intron 12 of the *Pltp* gene was amplified with Tks *Gflex* DNA polymerase (Takara Bio Inc., Shiga, Japan) using the following primers: an upper primer, GCGGCCGCGGGAATTCGTGCTAAGTTGACACTCCGGG, and a lower primer, ATCCAACGCGTTGGGAGCTCGGCGATGCCATTAACTACGC. The PCR products were subcloned into the pGEM-T easy vector (Promega, Madison, WI, USA) using the In-Fusion HD cloning kit (Takara Bio Inc.). Intron 12 in C3H-C and C3H-S was sequenced using a subcloning vector.

### Gene expression analysis via real-time PCR

Total RNA was isolated from the tissues using the TRI-reagent (Molecular Research Center, Inc., Cincinnati, OH, USA), according to the manufacturer’s protocol. To eliminate DNA contamination, the RNA was treated with DNase (TURBO DNA-free; Life Technologies Japan Ltd., Tokyo, Japan). Subsequently, cDNA was synthesized using a High-Capacity Reverse Transcription Kit (Life Technologies Japan Ltd.). Gene expression was quantified via real-time PCR using the StepOnePlus Real-Time PCR System with the Thunderbird SYBR qPCR Mix (Toyobo, Tokyo, Japan). Primers used for the SYBR Green Assay are listed in Supplementary Table S6. The mRNA levels were normalized to those of β-actin mRNA.

### Rapid amplification of cDNA 3′-ends (3′-RACE)

Total RNA was isolated from the lungs of C3H-C and C3H-S mice. mRNA from the total RNA was purified using the Oligotex-dT30 < Super > mRNA Purification Kit (Takara Bio Inc.). cDNA synthesis was performed using the 3′-Full RACE Core Kit with oligo dT-3 site adaptor primers (Takara Bio Inc.). Further, 3′RACE for defining PLTP transcript variants was performed using 1st PCR primer on exon 10 (F:GCCACCTACTTTGGGAGCAT), 2nd PCR primer on exon 12 (F:GTGCTAAGTTGACACTCCGGG), and 3 sites adaptor primer. The 3′-RACE products were subcloned and sequenced.

### Statistical analysis

All data are expressed as mean $\pm$ SD. Normality tests were performed for all data by Shapiro–Wilk test. To compare the mean of two groups, Welch’s test was used. To compare the mean of three groups with equal variances, one-way ANOVA followed by Tukey’s test were performed. To compare the mean of three groups with unequal variances, Welch’s ANOVA followed by Dunnett’s T3 comparison test were performed. When data from three groups was deviated significantly from normality, Kruskal–Wallis’s test followed by Dunn’s multiple comparison. The Spearman’s correlations for each pair of serum lipids concentration and single regressions analyses between PLTP mRNA and other traits were performed. These statistical analyses were performed using GraphPad Prism 9 (GraphPad Software, San Diego, CA, USA).

To determine the strongest locus associated with serum lipids concentrations, the correlation between each allele at loci on chromosome 2 and serum lipids concentrations (TG, TC, HDL-C and PL) of each mouse were analyzed. Statistical analyses for data of F2 mice were performed using JMP Pro version 13.2.0 software (SAS Institute Japan Ltd., Tokyo, Japan).

### Supplementary Information

Supplementary Information.

## Data Availability

Genome sequencing data were submitted to the SRA (Accession No. PRJNA827968). The ERV-inserted DNA sequence data obtained using the subcloning vector of intron 12 of the *Pltp* gene in the C3H-S mouse have been deposited in DDBJ under the accession number LC726248. The other datasets used and/or analyzed during the current study are available from the corresponding author upon reasonable request.
