# Supplementary material for: C3H/HeNSlc mouse with low phospholipid transfer protein expression showed dyslipidemia
Source: Sci Rep. 2023 Aug 24;13:13813. doi: 10.1038/s41598-023-40917-9 (PMC10449841; doi:10.1038/s41598-023-40917-9)

Supplementary table S1

The values of serum lipids in each genotype in male (C3H-S × C3H-C)F2 mice.

Marker (position)	Trait (mg/dL)	(C3H-S × C3H-C)F2		
		C3H-C/C3H-C	C3H-C/C3H-S	C3H-S/C3H-S
SNP_28.71 (28.71 Mb)		n = 14	n = 18	n = 9
	TG	163 ± 96	136 ± 63	**191 ± 93**
	TC	80 ± 27	**81 ± 20**	74 ± 24
	HDL-C	**70 ± 16**	69 ± 12	69 ± 15
	PL	248 ± 60	**265 ± 36**	262 ± 30
SNP_79.42 (79.42 Mb)		n = 13	n = 20	n = 8
	TG	122 ± 62 [a]	142 ± 65 [a]	**253 ± 89** [b]
	TC	**85 ± 23**	80 ± 19	65 ± 29
	HDL-C	**71 ± 12**	**71 ± 12**	63 ± 19
	PL	263 ± 56	**265 ± 35**	236 ± 44
SNP_112.68 (112.68 Mb)		n = 13	n = 20	n = 7
	TG	103 ± 38 [a]	160 ± 65 [a]	**250 ± 113** [b]
	TC	**89 ± 19** [a]	82 ± 22 [a]	53 ± 16 [b]
	HDL-C	**72 ± 10**	71 ± 13	61 ± 20
	PL	**272 ± 56**	261 ± 33	228 ± 41
SNP_163.08 (163.08 Mb)		n = 12	n = 21	n = 8
	TG	111 ± 36 [a]	130 ± 49 [a]	**299 ± 43** [b]
	TC	**94 ± 15** [a]	84 ± 18 [a]	44 ± 3 [b]
	HDL-C	**74 ± 10** [a]	**74 ± 12** [a]	52 ± 6 [b]
	PL	267 ± 56 [a]	**275 ± 21** [a]	204 ± 27 [b]
SNP_168.52 (168.52 Mb)		n = 11	n = 22	n = 8
	TG	107 ± 34 [a]	131 ± 49 [a]	**299 ± 43** [b]
	TC	**93 ± 15** [a]	85 ± 18 [a]	44 ± 3 [b]
	HDL-C	73 ± 10 [a]	**74 ± 12** [a]	52 ± 6 [b]
	PL	266 ± 59 [a]	**275 ± 20** [a]	204 ± 27 [b]

Mean ± SD. Underline indicates the maximum values of each trait at the respective marker. (C3H-S × C3H-C)F2 mice (n=41). [a,b] Means without common letters are significantly different by Tukey's (parametric) or Dunn's (non-parametric) multiple comparison test (*P*<0.05).

Supplementary table S2

The coefficient of determinations between the chromosomal markers and serum lipids concentrations in the (C3H-S × C3H-C)F2 mice.

Marker (position)	Trait	Coefficient of determination (R^2)	*P*-value
SNP_28.71 (28.71 Mb)	TG	0.069	2.56e-1
	TC	0.012	7.99e-1
	HDL-C	0.001	9.75e-1
	PL	0.029	5.66e-1
SNP_79.42 (79.42 Mb)	TG	0.342	4.00e-4
	TC	0.092	1.61E-1
	HDL-C	0.062	3.00E-1
	PL	0.064	2.85E-1
SNP_112.68 (112.68 Mb)	TG	0.358	2.16e-4
	TC	0.284	1.76e-3
	HDL-C	0.080	2.07e-1
	PL	0.117	9.45e-2
SNP_163.08 (163.08 Mb)	TG	0.727	1.96e-11
	TC	**0.592**	**4.01e-8**
	HDL-C	0.423	2.90e-5
	PL	0.380	1.13e-4
SNP_168.52 (168.52 Mb)	TG	**0.733**	**1.28e-11**
	TC	0.578	7.28e-8
	HDL-C	**0.423**	**2.89e-5**
	PL	**0.381**	**1.09e-4**

Underline indicates the strongest correlation of each trait. (C3H-S × C3H-C)F2 mice (n=41).

Supplementary table S3

The microsatellite markers used for genotyping of High-TG group and Low-TG group in F2 male mice.

Primers		Forward and Reverse primer (5'→3') [a]	Position (cM)[a]	Physical position (bp)[a]
D1Mit173	F:<br>R:	AATCCTTCAATTATCTCCAATAGGC<br>TTAAGTAGCAGGGTTCTGTTAGGG	20.1	41503591-<br>41503736
D1Mit14	F:<br>R:	GCCAGACAGGGCTACATTGT<br>AGACTGAACTCTGGCCTCCA	67.7	156774467-<br>156774646
D1Mit17	F:<br>R:	GTGTCTGCCTTTGCACCTTT<br>CTGCTGTCTTTCCATCCACA	95	189571254-<br>189571420
D2Mit156	F:<br>R:	ACTGGGGAGACTAAATGGGG<br>ACTCTTCCATGCAACCGATT	31.7	57081653-<br>57081799
D2Mit62	F:<br>R:	GGATACCGTTTGGAAAGTAAACC<br>GCAAGAAGCACAGGAGGC	59.3	118112449-<br>118112609
D2Mit226	F:<br>R:	TTTTTGCAACTTTGTTAAGAATTCC<br>AAAACACCCTCCCACCCTT	84.1	163194446-<br>163194546
D3Mit333	F:<br>R:	CTCCCTCCCTTCCTCCTTC<br>ACAAAAGCAGAAGACTGATCCC	20.3	44707730-<br>44707852
D3Mit11	F:<br>R:	CCAACCACAGTAACACATGT<br>TGGAGACCAATGCGAACAAC	43.7	100556675-<br>100556821
D3Mit116	F:<br>R:	TCACTGCCCATCTTTGTAACC<br>CCCAGAGACCCGGAATAGAA	79.2	154541602-<br>154541860
D4Mit236	F:<br>R:	TCTTAGCATGCTTACCGCCT<br>GGCCCGTAGGATGACTGTC	20.0	39111944-<br>39112145
D4Mit15	F:<br>R:	AGGAATACTGAATGTGGACTTTCC<br>TCCCTTGATTAACAGAAGACCTG	43.2	91556462-<br>91556746
D4Mit204	F:<br>R:	CTGCTGCAGCGATTCTCTC<br>TCAGGCACCTAAGTACATGTGC	66.3	133427367-<br>133427471
D5Mit349	F:<br>R:	TCCAGTTCTCATAGATGTCTAAGCC<br>CTTCTGTGTATCTACACCACTGGG	12	24969162-<br>24969301
D5Mit254	F:<br>R:	GTGCAGGCCTGAATTGAAAT<br>CAAAGTGCCTGTGCATGTG	30.6	55230992-<br>55231125
D5Mit239	F:<br>R:	ATTGCAGACATAAAGGATATTTTGG<br>GCCAGCCTGGCTTACATAAG	52.2	107413140-<br>107413285
D6Mit1	F:<br>R:	GGCACATTTGCCTTTGTTTT<br>TCTCCTATCTCTCCACCTTTTCC	6.7	15144274-<br>15144496
D6Mit384	F:<br>R:	AATGCTTTATATGCAAACTACTCTCTC<br>GAATATAGCAAGACAAGGGAGACA	27.4	55199866-<br>55199990
D6Mit254	F:<br>R:	AGTGTCCCTAGGGGGTGG<br>GGGGCCTTAGAGGTAGCAAC	59.3	125356646-<br>125356785
D7Mit229	F:<br>R:	GGTTCTCTTTCCTTGTTTGCC<br>TACTGGTTACATCTGGTGGGTG	29.4	45686980-<br>45687102
D7Mit351	F:<br>R:	TCATCCTAGGATTCTCTGCACA<br>CTTTGTGAGGTGTTCTCTCCG	52.9	96901062-<br>96901168
D7Mit66	F:<br>R:	TTCACTCCCAGCCAGTCTCT<br>TAACCAGGAAACACACGAACC	64.3	119885996-<br>119886157
D8Mit294	F:<br>R:	AAGACAGCACACTTTTAGTTAGTGTG<br>AGGCTAGTGATGGTGGACTCA	23.9	39405411-<br>39405520
D8Mit249	F:<br>R:	AAACTCACACACACAGAGACAACA<br>GCCCCAGTGTGACTAAGGAG	38.6	80939547-<br>80939696
D8Mit42	F:<br>R:	ATTATCCCAAGGTGGCCTTC<br>AGGGAACTCACGTTCACACC	74.5	126552317-<br>126552465
D9Mit22	F:<br>R:	ATTGCATAACACCCCCACAT<br>CAGTGCTTAACTGCTCAAATGC	26.8	49508998-<br>49509211
D9Mit10	F:<br>R:	TAACCAACCCTTCAAGGCAC<br>AATCCTTGGCTGAAGGGAAT	47.6	89977005-<br>89977152
D9Mit18	F:<br>R:	TCACTGTAGCCCAGAGCAGT<br>CCTGTTGTCAACACCTGATG	71.5	120289445-<br>120289624

Supplementary table S3 （Continued）

D10Mit194	F:<br>R:	GATTGTTTGTAAAGACATGATCACG<br>AGATGTGGAATAGGAAGTATGATCG	24.5	46844307-<br>46844387
D10Mit12	F:<br>R:	ATGTCCAAAACACCAGCCAG<br>GGAAGTGATGGAGCTCTGTT	51.2	99473556-<br>99473795
D11Mit229	F:<br>R:	TGTTTGCTTGGTTTTGTTTTG<br>ATCCCGGTTTTACATCAGACA	15.6	25956097-<br>25956218
D11Mit140	F:<br>R:	GCATTTACTTGATTGATTGTTTGC<br>ACCCAATGCCTGCCTCTAC	32.1	54203495-<br>54203622
D11Mit14	F:<br>R:	CCACTTAGTATATCTTGTCC<br>GCATGACTTGGCCTATCACC	62.4	98683655-<br>98683810
D12Mit84	F:<br>R:	ATAAGTTAGGGGAAATCACTGGG<br>GGTGTGGCTTTCCCAAACTA	10.0	27570643-<br>27570810
D12Mit52	F:<br>R:	CCATCTTCTGGCATTTTGCT<br>AGACAGGAGGGTCCCAAAGT	34.1	76984294-<br>76984429
D12Mit132	F:<br>R:	CCATATACATTTCTAACACCCTTGC<br>AGAACTTACTTCTAGTGGAGACAATGC	57.7	106972772-<br>106972901
D13Mit3	F:<br>R:	TCAGGCTCATCCCAGATACC<br>TTTTGCAGAGAACACACACC	7.3	20431342-<br>20431502
D13Mit13	F:<br>R:	CTGTGGTAAGTCCAGATTTG<br>GGAAAGAGTAGGAAGATGCC	30.1	56481436-<br>56481584
D13Mit148	F:<br>R:	TGCTTGTGCTCATGCATACA<br>AAAGGAAGGTGGCAAGTAATAGG	59.7	108828769-<br>108828903
D14Mit60	F:<br>R:	AGGCTGCCCATAAAAGGG<br>GTTTGTGCTAATGTTCTCATCTGG	24.6	47097793-<br>47097924
D14Mit125	F:<br>R:	GTTGAGGTCCCACTGCAAAT<br>TTGAAGGAGATATATCACTCTGTGTG	44.2	87626332-<br>87626490
D14Mit266	F:<br>R:	ATGCACAGGATTGATCTGCA<br>AGCATGACCTAAATAATGAGACCC	64.9	121032245-<br>121032392
D15Mit130	F:<br>R:	CATATTTTGCAATTTTTAGTAATAGGC<br>CAACACAGAAATAAAAGTGAGAGAGG	8.2	20807266-<br>20807449
D15Mit63	F:<br>R:	ACCAATGATCGTTGATGCCT<br>TAATTTCACACTAGCAAAACCAAA	29	65245184-<br>65245328
D15Mit161	F:<br>R:	TCTGTTTTGTTTGTTCGTTTGC<br>TAAAATCTCCCTGTATACAAGTCTGTG	52.8	97011059-<br>97011157
D16Mit12	F:<br>R:	GAACTCAGTAAGCTCTCTATGCCC<br>GGAGGACTAGCAGGCTAGAGC	27.0	39118021-<br>39118212
D16Mit152	F:<br>R:	AGAGACCTCTGGGGTGGG<br>TTCAAGATAGACTATTCTGGAAAAAGC	48.2	85803834-<br>85803938
D17Mit23	F:<br>R:	TCGAGCTGGTTGAACGAAC<br>CGGGAAAGCATGGAATTTAA	15.4	29492190-<br>29492323
D17Mit123	F:<br>R:	CACAAGGAGGGAGCCTGTAG<br>CACCGTAAGAGTCTAATAATAAGGGG	60.7	93199619-<br>93199751
D18Mit15	F:<br>R:	CAGACTTCATAGCAACACCCTG<br>TAACATGAAAACAGAAACAGCCA	18.8	35006651-<br>35006806
D18Mit184	F:<br>R:	CACACATGTGTAGGTAGGTAGGTAGG<br>CGCACAAGGACTACTGAAACA	39.7	66819243-<br>66819414
D18Mit4	F:<br>R:	ACTGTTGCTGGGGAATGG<br>CCAAGTTCAAAGCTGCTGG	57.5	84126264-<br>84126470
D19Mit56	F:<br>R:	CTGAATGTGTATGTGTGCAAGTATG<br>ATTATGAATTCAAGACTAGCCTAGGA	6.2	9933199-<br>9933333
D19Mit11	F:<br>R:	TCAAAGTCAAGGTGGGCAG<br>ACTTTCCAGATGTTGGGCAC	36.3	42394609-<br>42394755
DXMit166	F:<br>R:	GAGATAAACCTGACTAACCCTTTCC<br>GGATTTTCCCAAAAAAGAAACC	28.3	51683498-<br>51683611
DXMit79	F:<br>R:	AGTCTGCCTTCTCTTTCTGTATCC<br>TGAAACTATTCCAACATTATTCTTGG	53.2	130863870-<br>130864007

[a], Primer sequence, cM and physical position were obtained from the Mouse Genome Informatics (http://www.informatics.jax.org/).

Supplementary table S4

The primers and restriction enzymes used for genotyping of (C3H-S×B6)F2 male mice on chromosome 2.

Primers		Forward and Reverse primer (5'→3') [a]	Position (cM)[a]	Physical position (bp)[a]	Restriction enzyme[b]
D2Mit276	F:<br>R:	AGGAAGCACCAAGTCTGCAT<br>CACTAAACCCTACATGGTACAGTCC	59.4	118,442,067-<br>118,442,203	-
D2Mit258	F:<br>R:	ACCTCACTCACCTCTCAGAAATG<br>TGCTTATGCACAAAGCCTTG	63.2	130,407,617-<br>130,407,721	-
rs27257722	F:<br>R:	CTGTGTTCCCGTGTTCCAG<br>GCTTGGTTTCTGGCCCTACAG		145,973,647	XspI
rs27294950	F:<br>R:	TGACTCCGGCTTGCTTACTT<br>GCAGCACCCCAGATATCAAT		147,068,748	AccI
rs29500584	F:<br>R:	TTCATGGTTGGTTGAGTCCA<br>CGATTTCTGGCTCAGCTCTC		153,628,450	SalI
rs27325969	F:<br>R:	TCAAAAAGTCTTGTTTTTCTGCAA<br>TCTGCTTGCTTGTTCTGGTG		155,212,992	PstI
D2Mit226	F:<br>R:	TTTTTGCAACTTTGTTAAGAATTCC<br>AAAACACCCTCCCACCCTT	84.1	163,194,446-<br>163,194,546	-
rs13459398	F:<br>R:	GGCTCTCCTCTCTTTGCAGG<br>ACTGTGTCCAGGAGGGAGTT		164,852,656	HpyCH4IV
rs27295296	F:<br>R:	CAAAAGGGAGCGAAACAGAG<br>GCTCTGGTTTGAGGAGATGG		166,077,467	AccI
rs51710768	F:<br>R:	TACCGCCTTATGCTTGTCCT<br>GGTTTTGCGAACCTTGATGT		173,527,895	SacI
rs27693033	F:<br>R:	AGCCCCTATGCTGGTACCTT<br>GAGGCTCTGGGCTCCTAATC		181,206,785	EcoRV

[a], Primer sequence, cM and physical position were obtained from the Mouse Genome Informatics (http://www.informatics.jax.org/).

[b], Restriction enzyme was used for the determination of the SNPs.

Supplementary table S5

Primers used for genotyping of male (C3H-S×C3H-C) F2 mice.

marker		Forward and Reverse primer (5'→3')	bp	Restriction Enzyme
SNP _28.71 Mb	F:<br>R:	CCCTGATGGATTTGGCTGGT<br>CTGGGGACATTTCCCAGCAT	28,707,436	AvaII
SNP _79.42 Mb	F:<br>R:	AACTCCTCCTAGCACACCCT<br>CAAATCTTCCACTGGCCCCT	79,420,407	MspI
SNP _112.68 Mb	F:<br>R:	TGCATGTGCACTGGCTCTTA<br>TCCTCTGTTAGCTTGGCAGC	112,681,249	RsaI
SNP _163.08 Mb	F:<br>R:	AAGAGGCCTGAACTGCCTTC<br>GGTTCTGTCAGGTGAGGTCG	163,084,934	AciI
SNP _164.12 Mb	F:<br>R:	GGAAGAGCGTTTTGGAAGGT<br>AAAACATCCTTGGGGAAAACTCAG	164,123,955	SphI
SNP _168.53 Mb	F:<br>R:	CCTGACTTGGAATGGCAGTCT<br>GCTGATCATGGAACCCGTCG	168,528,450	HpyCH$_4$IV

Supplementary table S6
The primers for SYBR real-time PCR.

Primers		Forward and Reverse primer (5'→3')	Accession No.	position
β-actin	F:	AGATGACCCAGATCATGTTTGAGA	NM_007393.3	462-526
	R:	CACAGCCTGGATGGCTACGT		
Pltp	F:	CCGAGTGACCTGGACATGCT	NM_0011125.2	1022-1086
	R:	CTCGGACTCAGGAGAACAATGC		

Supplementary Figure Legends

Supplementary Fig. S1

Genotyping using pooled genomic DNA of F2 mice at D2Mit62 and D2Mit226.

(A) To detect the chromosome region associated with serum TG concentration, PCR were performed by using pooled DNA of high-TG group (top 15 mice of serum TG concentration) or low-TG group (bottom 15 mice of serum TG concentration) in F2 mice. By using microsatellite markers, the apparent differences in PCR product size of electrophoresis between high-TG group and low-TG group were detected at D2Mit226 on chromosome 2. The lanes of B6, F1 and C3H-S show control genomic DNA. At D2Mit226, the lanes of high-TG show that most of top 15 mice have C3H-S allele. In contrast, the lanes of low-TG show that most of bottom 15 mice have B6 allele. **(B)** Original gel in Fig. S1A.

Supplementary Fig. S2

The correlation of serum lipids concentrations in (C3H-S × C3H-C)F2 mice.

(A) TG vs. TC, **(B)** TG vs. HDL-C, **(C)** TG vs. PL, **(D)** TC vs. HDL-C, **(E)** TC vs. PL, **(F)** HDL-C vs. PL. (C3H-S × C3H-C)F2 mice (n=41). Spearman's correlations was performed for each pair of the serum lipids. NS, not significant.

Supplementary Fig. S3

The regression of PLTP mRNA levels for serum lipids in (C3H-S × C3H-C)F2 mice.

(A-D) Sigle linear regression of Epi-fat PLTP mRNA levels for serum lipids in (C3H-S × C3H-C)F2 mice (n=41). The mRNA levels of C3H-C/C3H-C genotype at SNP_161.12 Mb was set to 1. NS, not significant.

Supplementary Fig. S4

The sequences of Pltp transcripts (S1 and S2 in Fig.6E) and predicted amino acid.

(A) S1, **(B)** S2. Exon 12 was shown in green marker and intron 12 was shown in gray letters. Target site duplication (TSD) was shown as yellow line. LTR (383) are shown in red letters (S1; 323 bp, S2; 81 bp) and the gag-pol protein is shown in blue.

Supplementary Fig. S5

The original agarose gels electrophoresis of Fig. 6.

(A) Original gel of Fig. 6A. **(B)** Original gel of Fig. 6D.

Supplementary Fig. S1

(A)

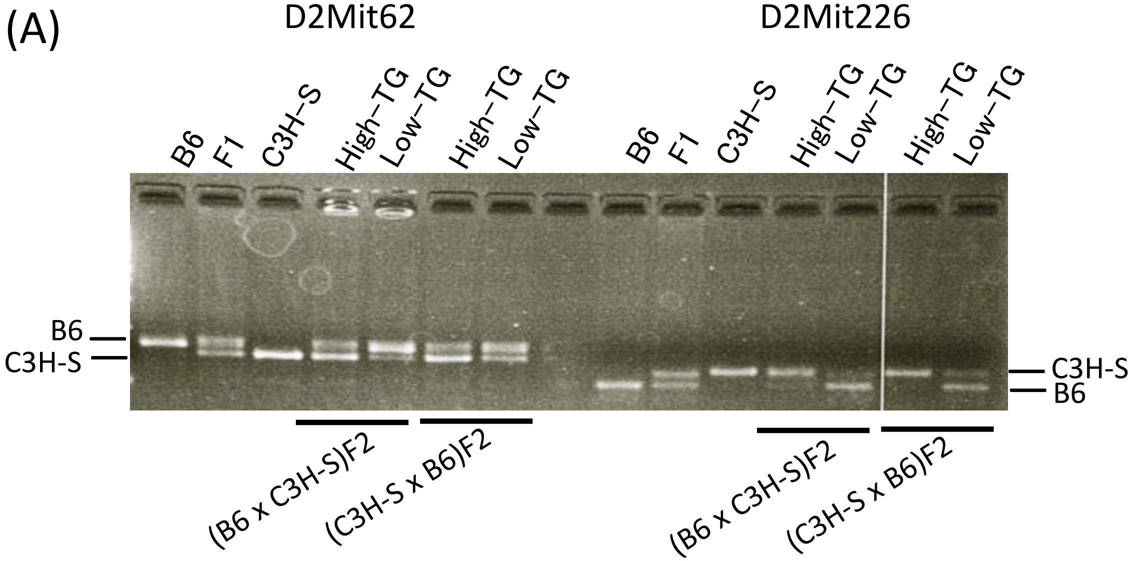

(B)

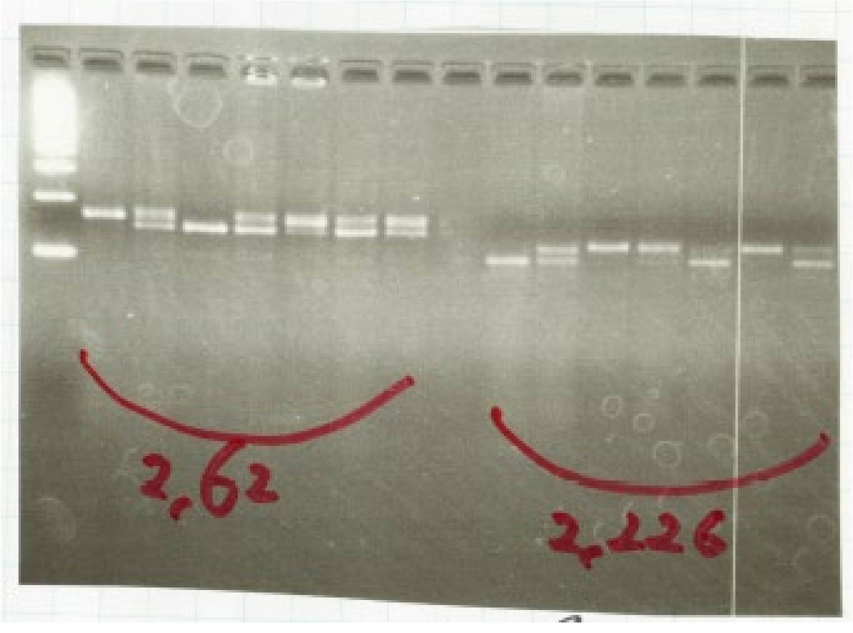

Supplementary Fig. S2

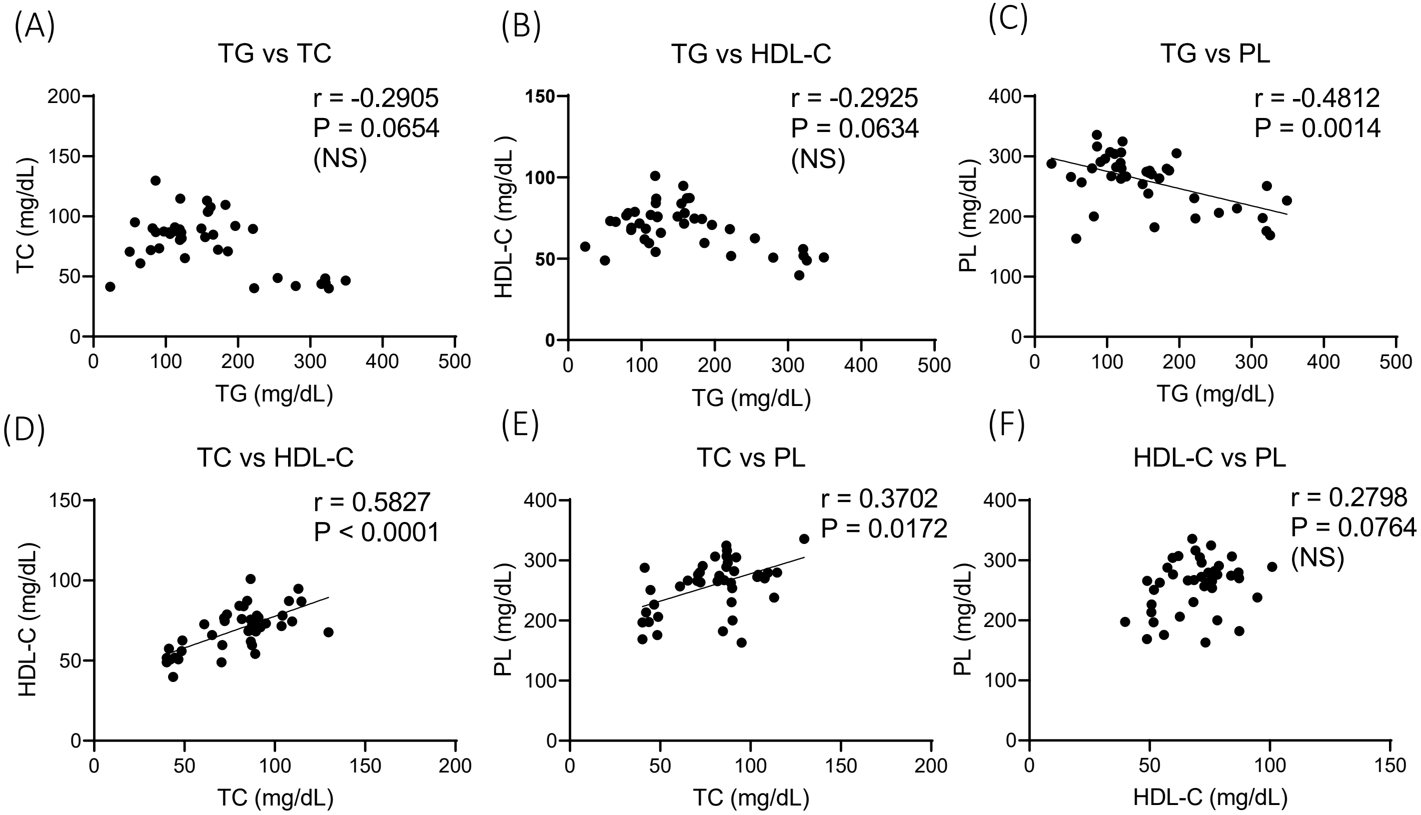

Supplementary Fig. S3

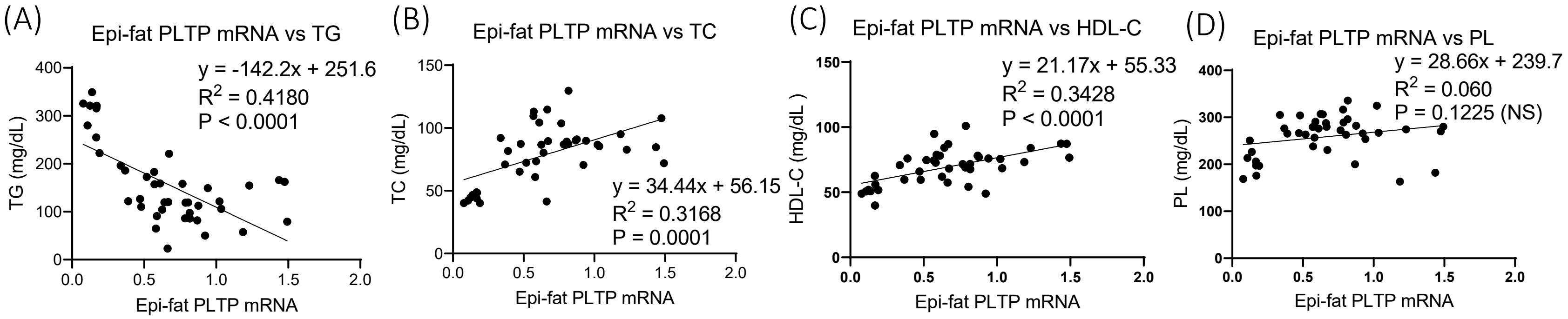

Supplementary Fig. S4

(A)

```
        10         20         30         40         50         60
GTGCTAAGTT GACACTCCGG GGCAAGGCGC TGCGAGTGAA GCTGGACCTT CGAAGGTATT
  A  K  L   T  L  R   G  K  A  L   R  V  K   L  D  L   R  R  Y  S

        70         80         90        100        110        120
CCCAGCCTTA GCATCTCTAG GATAGTGGTA CCTGCTCAAC AGAACTGCAG GGCTAAAGCT
  Q  P  *

       130        140        150        160        170        180
GGGCTAGAAC TCCTGTAAGG CTATCCAGAG TTATATAGTG AGACTATGCT GAGAGAGAGA

       190        200        210        220        230        240
GAAAGAGAGA GAGAGAGAGA GAGCGCGAGA GAGAGCGCGA GTAAACATGA CACATACCTT

       250        260        270        280        290        300
TAATCCCGGG GATGGTGACC AGAGATAGGT GGATCCTTGC AATCTTAGAG GAGAGCTAGC

       310        320        330        340        350        360
CTGCCCTATG TGGTGAATTT CCAGGCTGAT GAAACACCAG TCTCAAACAA AAGGAGGGAA

       370        380        390        400        410        420
GGGCTATGAA CAAATACCCT GAATGCTGTC CTCTAACCTT TACATGCATG CCAAGGACAC

       430        440        450        460        470        480
ACGTACCTGC ACCTACACAT CCAAAATCTA TCTGACCATG TAGCCAAAGA CGATCCTGCT

       490        500        510        520        530        540
TGCATCTCCA GTGTGCTGGG ATTACAGGCA CATACTACCA TGTCCTGTCT ATATAATGCT

       550        560        570        580        590        600
CAGGGACGGA ACCCAGAGCC TAGTGCTGTG GGAAGCCGCC CCCACATTCG CCGTCACAAG

       610        620        630        640        650        660
ATGGCGCTGA CATCCTGTGT TCTAAGTTGG TAAACAAATA ATCTGCGCAT GAGCCAAGGG

       670        680        690        700        710        720
TATTTACGAC CACTTGTACT CTGTTTTTCC CGTGAACGTC AGCTCGGCCA TGGGCTGCAG

       730        740        750        760        770        780
CCAATCAGGG AGTGATGCGC CCTAGGCAAT GGTTGTTCTC TTTAAATAGA AGGGGTTTCG

       790        800        810        820        830        840
TTTTTCTCTC TCTCTTGCTT CTTGCTCTCT CTTGCTTCCC TCTCTTGCTT CTTGCTCTCT

       850        860        870        880        890        900
CTTGCTTCTT GCTCTCTTTC CTGAAGATGT AAGAATAAAG CTTTGTCAGA AAAAAAAAAA
```

(B)

```
        10         20         30         40         50         60
GTGCTAAGTT GACACTCCGG GGCAAGGCGC TGCGAGTGAA GCTGGACCTT CGAAGATGTA
  A  K  L   T  L  R   G  K  A  L   R  V  K   L  D  L   R  R  C  K

        70         80         90        100        110        120
AGAATAAAGC TTTGTCGCAG AAGATTCTGG TCTGTGGTGT TCTTCCTGGC CGGTCGTGAG
  N  K  A   L  S  Q   K  I  L  V   C  G  V   L  P  G   R  S  *

       130        140        150        160        170        180
AACGCGTCGA ATAACAATTG GTGCCGAATT CCGGGACGAG AAAATCCGGG ACGAGAAAAA

       190        200        210        220        230        240
ACTCCGGACT GGCGCAGGAG GGATACCTCA TTCCAGAACC AGAACTGCGA ATCAAGGTTA

       250        260        270        280        290        300
TAAGGTTCCC GTAACACAGA CTGTTGAGAA GGATTCAACT GCCGAATTCA GAACTCATCA

       310        320        330        340        350        360
GCTGGGGAAC GACGGTGATA AAGGTTCCCG TAAAGCAGAC TGTTAAGAAG GATTCAACTG

       370        380        390        400        410        420
TATGAATTCA GAACTTTTCA GCTGGGGAAC GAGGTAAGTC TGATCTTGAA CTTTCTAAGG

       430        440        450        460        470        480
AAATTCAAGA CAGTCTATCA GAAGTAAAGT GGAAAATGGC TTTACAAGTT ATGTTTGGCC

       490        500        510        520        530        540
TTAAATTTTT TCTAGTGTTA GAAGCCCTTT TGTTCCTTTT CACATGTTAT ATAGTGCTTA

       550        560        570        580        590        600
AGATAGGGCT GAAAATTCTA GAGGAAATTC AGGACAGTCT ATCAGAAGTA AAGCGGGGAG

       610        620        630        640        650        660
AGAGAGTAGG AGCAAGGAGA AACGGTAAGT ATACAGGCCT TTCCAAGGGT CTTGAACCCG

       670        680        690        700        710        720
AGGAAAAGTT AAGGTCAGGT AGGAATACCT GGGGAGAGAT TAGAAGGAAG AGATTAGAAG

       730        740
CAAAAAAAAA AAAAAA
```

Supplementary Fig. S5

(A) Original gel in Fig. 6A

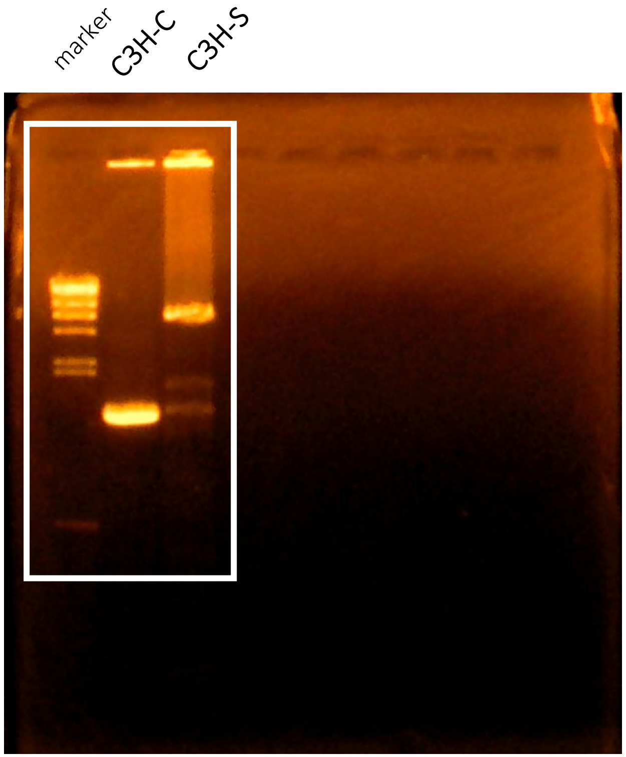

(B) Original gel in Fig. 6D

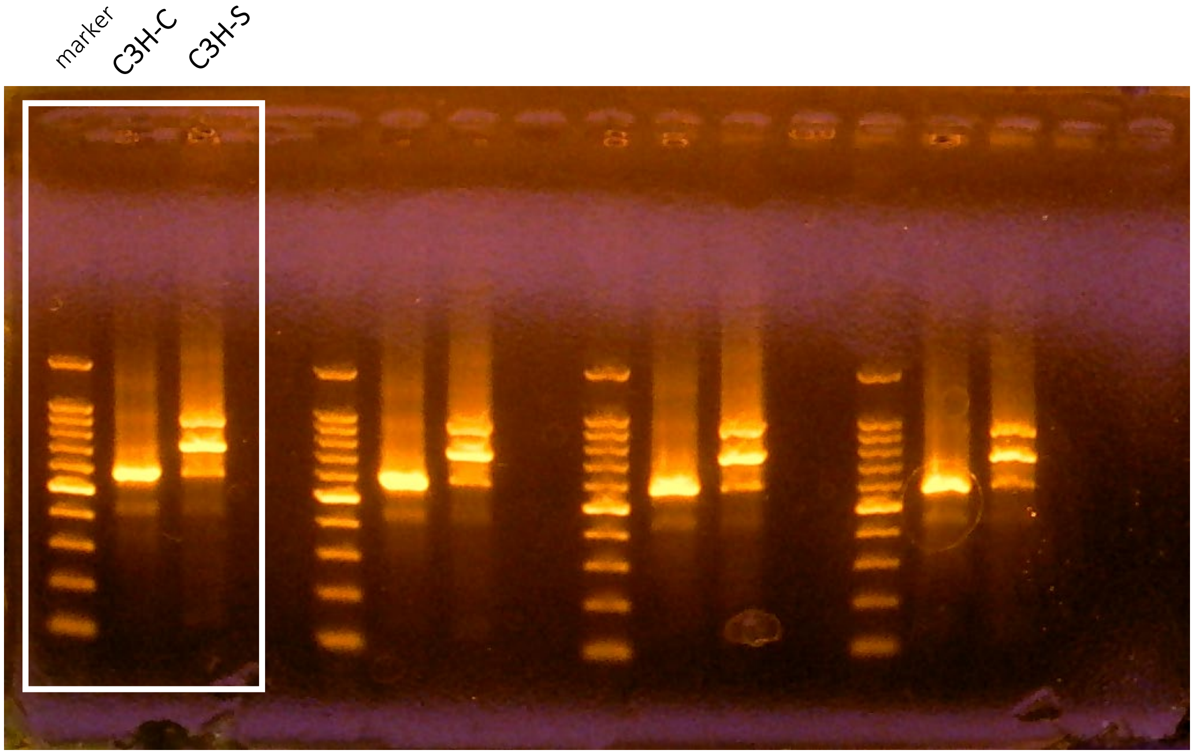

Supplement: Supplementary file 1 — Supplementary Information. [file 41598_2023_40917_MOESM1_ESM.pdf]
